# Improper wound treatment and delay of rabies post-exposure prophylaxis of animal bite victims in China: Prevalence and determinants

**DOI:** 10.1371/journal.pntd.0005663

**Published:** 2017-07-10

**Authors:** Qiaoyan Liu, Xiaojun Wang, Bing Liu, Yanhong Gong, Naomie Mkandawire, Wenzhen Li, Wenning Fu, Liqing Li, Yong Gan, Jun Shi, Bin Shi, Junan Liu, Shiyi Cao, Zuxun Lu

**Affiliations:** 1 School of Public Health, Tongji Medical College, Huazhong University of Science and Technology, Wuhan, Hubei, China; 2 Center of Health Administration and Development Studies, Hubei University of Medicine, Shiyan, Hubei, China; 3 School of Economics and Management, Jiangxi Science and Technology Normal University, Nanchang, Jiangxi, China; 4 Department of Orthopedics, Shiyan Traditional Chinese Medicine Hospital, Shiyan, Hubei, China; 5 Xinhua Street Community health center of Jianghan District, Wuhan, Hubei, China; Wistar Institute, UNITED STATES

## Abstract

**Background:**

Rabies is invariably a fatal disease. Appropriate wound treatment and prompt rabies post-exposure prophylaxis (PEP) are of great importance to rabies prevention. The objective of this study was to investigate the prevalence and influencing factors of improper wound treatment and delay of rabies PEP after an animal bite in Wuhan, China.

**Methodology:**

This cross-sectional study was conducted among animal bite victims visiting rabies prevention clinics (RPCs). We selected respondents by a multistage sampling technique. A face-to-face interview was conducted to investigate whether the wound was treated properly and the time disparity between injury and attendance to the RPCs. Determinants of improper wound treatment and delay of rabies PEP were identified by a stepwise multivariate logistic regression analysis.

**Principal findings:**

In total, 1,015 animal bite victims (564 women and 451 men) responded to the questionnaire, and the response rate was 93.98%. Overall, 81.2% of animal bite victims treated their wounds improperly after suspected rabies exposure, and 35.3% of animal bite victims delayed the initiation of PEP. Males (OR = 1.871, 95% CI: 1.318–2.656), residents without college education (OR = 1.698, 95% CI: 1.203–2.396), participants liking to play with animals (OR = 1.554, 95% CI: 1.089–2.216), and people who knew the fatality of rabies (OR = 1.577, 95% CI: 1.096–2.270), were more likely to treat wounds improperly after an animal bite. Patients aged 15–44 years (OR = 2.324, 95% CI: 1.457–3.707), who were bitten or scratched by a domestic animal (OR = 1.696, 95% CI: 1.103–2.608) and people who knew the incubation period of rabies (OR = 1.844, 95% CI: 1.279–2.659) were inclined to delay the initiation of PEP.

**Conclusions:**

Our investigation shows that improper wound treatment and delayed PEP is common among animal bite victims, although RPCs is in close proximity and PEP is affordable. The lack of knowledge and poor awareness might be the main reason for improper PEP. Educational programs and awareness raising campaigns should be a priority to prevent rabies, especially targeting males, the less educated and those aged 15–44 years.

## Author summary

Although the incidence of animal bites is increasing in China, residents’ knowledge about appropriate wound treatment and prompt PEP is insufficient. A face-to-face interview was conducted to investigate whether the wound was treated properly and to determine the time disparity between injury and consultation to the RPCs among animal bite victims. We discovered that a minority of people treated wounds appropriately, and more than one-third of participants delayed the initiation of PEP. A stepwise multivariable logistic regression analysis was used to identify the influencing factors of improper wound treatment and the delay of rabies PEP. Males (OR = 1.871, 95% CI: 1.318–2.656), residents without college education (OR = 1.698, 95% CI: 1.203–2.396), respondents liking to play with animals (OR = 1.554, 95% CI: 1.089–2.216), and people who knew the fatality of rabies (OR = 1.577, 95% CI: 1.096–2.270), were likely to treat wounds improperly after an animal bite. Participants aged 15–44 years (OR = 2.324, 95% CI: 1.457–3.707), residents bitten or scratched by a domestic animal (OR = 1.696, 95% CI: 1.103–2.608), and people who knew the incubation period of rabies (OR = 1.844, 95% CI: 1.279–2.659) tended to delay the initiation of PEP. The results also showed that knowledge of rabies among residents is insufficient. These findings highlight the urgent need for public educational and awareness-raising programs that would improve appropriate wound treatment and prompt PEP to prevent rabies-related deaths.

## Introduction

Rabies is a fatal infectious disease, which causes severe neurological symptoms that unavoidably result in death. Although human rabies is currently untreatable [1], appropriate post-exposure prophylaxis (PEP) can entirely prevent rabies [2,3]. Such PEP, which consists of local treatment of the wound, followed by vaccine (with or without rabies immune globulin [RIG] depending upon the type of exposure) should be initiated immediately after a suspected rabid bite. Recommended first-aid procedures include immediate and thorough flushing and washing of the wound for a minimum of 15 minutes with soap and water, as well as disinfecting the wound with detergent or other substances of proven lethal effect on the rabies virus. Appropriate wound cleansing and disinfection can prevent one-third of rabies infections [4–6]. Modern cell-culture vaccines utilized in combination with RIG are nearly 100% effective in preventing human deaths if inoculated promptly to rabies virus-exposed victims following appropriate wound management [7]. Unfortunately, it is inexcusable that more than 70,000 people die from rabies annually all over the world [8].

A growing body of research has shown that appropriate and prompt PEP are not well implemented by exposed victims in rabies endemic countries. Kabete [9] reported that only 7% of animal bite victims in Ethiopia washed the wound with soap and water as first aid, with only 53% of victims seeking PEP within the first 24 hours after the bite. A similarly low proportion of wound treatment has been reported in India, with 41% of animal injury victims initiating PEP within 48 hours [10,11]. Moreover, only 37.2% of animal bite cases in Iran received timely PEP (less than 6 hours) [12]. Gender [12], type of animal [12] and injury status [12] were associated with delay of initiation on PEP. The main reasons for delay of PEP included unaffordability of rabies vaccine and RIG [10,13], shortage of vaccines [14], long distance to the vaccination center [12,15], lack of RPCs and shortage of medical staff [14]. Such improper wound care and delayed PEP can result in a high incidence of rabies-associated mortality [16,17]. Most studies reported that human rabies cases were not treated with proper wound care, and few of them received prompt PEP [18,19]. The odds of suffering from rabies following exposure were therefore significantly higher for those who did not receive prompt PEP (OR = 17.33, 95% CI: 6.39–60.83) [16]. Inadequate wound care is one of the most commonly encountered causes of PEP failure [6].

China is the world’s largest producer and consumer of rabies vaccine, and it is ranked second in the world for the number of reported rabies cases [20]. From 1960 to 2014, China had reported a total of 120,913 human rabies cases, with a yearly average of 2,198 [21]. The number of domestic dogs and cats in China reached 150 million during 2013 [22], and it has been increasing by 10% yearly [23]. According to the statistics provided by the Ministry of Public Health of China in 2009, it was estimated that more than 40 million people were bitten or scratched annually in China. China’s high prevalence of animal bites makes rabies prevention an essential health priority. However, existing studies on the implementation of rabies PEP are mainly based on India [10] and African countries [16, 24], with related factors mostly being unaffordability and inaccessibility of the rabies vaccine. Luckily, effective anti-rabies biologics are available and affordable for Chinese animal bite victims. Hence, the above factors might not have the same influence on Chinese animal bite victims. A community-based study in Bhutan, where rabies PEP is free, showed that a knowledge gap might be the reason for an absence of PEP [25]. An examination on the level of rabies knowledge among rabies virus exposure victims may help in designing an appropriate and effective rabies prevention program for the public.

To bridge this data gap, the present study focused on PEP of animal bite victims. To our knowledge, this is the first reported study to identify potential risk factors for improper wound treatment and delayed PEP in China. In this study, we attempted to provide information on the determinants of improper and delayed PEP among animal bite victims, and to develop a practical and effective rabies prevention strategy by knowledge dissemination and raising awareness.

## Methods

### Study site

The present study was carried out between March 1 and May 31 2016 in Wuhan city, which is one of the seven biggest cities in China, with a resident population of 10.6 million. It is also one of the five largest pet cities in China, with more than 130,000 domestic animals [23, 26], most of which are dogs. The Wuhan Centers for Disease Prevention and Control reported that dogs injured more than 60,000 people yearly, and the annual consumption of rabies vaccine was more than 100,000 regimens.

### Ethics statement

The Research Ethics Committee in Tongji Medical College of Huazhong University of Science and Technology approved the study. The methods of the present study were implemented in accordance with the approved protocols. All participants read the purpose statement of the investigation and signed informed consents. Written informed consent was attained from all the guardians of minors (under 18 years old) after an explanation of the study purpose. The present study was carried out in accordance with the approved protocol.

All data were anonymized and handled confidentially.

### Sampling procedure

A multistage sampling technique was used to select participants. Of the 15 districts in Wuhan city, three were selected by simple random sampling. Within each district, two RPCs were randomly selected and 180 eligible respondents were interviewed in each RPC. The last stage of selection of respondents within the clusters was non-random. As we know, cluster sampling is a method which could be used in the following situations: the population is concentrated in "natural" clusters (communities, schools, hospitals, etc.) or constructing a complete list of population elements is difficult. In the present study, as served by the same public health sector, the individuals in the same cluster share similar socio-demographic characteristic and knowledge of rabies. It is also hard to construct complete frames of animal bite victims. Therefore, cluster sampling is a suitable sampling method for our study. Of the 1,080 animal bite victims approached, 65 individuals did not complete the questionnaire and a total of 1,015 participants were interviewed (response rate of 93.98%) by experienced survey interviewers over a period of three months between March and May 2016. If a participant was younger than 15 years old, the guardian who accompanied the minor to the RPC was asked to complete the questionnaire instead.

### Data collection

The investigation was organized and coordinated by Huazhong University of Science and Technology and Wuhan Association of Community Health. According to the study protocol, Huazhong University of Science and Technology offered training to junior investigators who conducted the survey on animal bite victims seeking medical care in RPCs. The senior investigators checked the collected questionnaires daily to perform quality control. Data were entered double-blindly into the database by two different researchers using EpiData 3.0 to guarantee accuracy.

### Measures

The two dependent variables were: wound treatment is proper or wound treatment is improper; and the prompt initiation of PEP or the delay of initiation on PEP.

We enquired from respondents on what they did after animal bite. They were asked whether they squeezed the wound, cleaned the wound with water only, cleaned the wound with soap and water, disinfected the wound with detergent, bandaged the wound, did nothing, or took other actions. Response options were as follows: 1 = “squeezed the wound”, 2 = “flushed and cleaned the wound with water only”, 3 = “flushed and cleaned the wound with soap and water”, 4 = “disinfected the wound with detergent”, 5 = “bandaged the wound”, 6 = “did nothing”, 7 = “took other actions”. This variable was recorded so that 0 = “proper wound treatment (flushed and cleaned with soap and water or water only, as well as disinfected the wound with detergent)”, and 1 = “improper wound treatment”. Refused (n = 53) responses were excluded.

We investigated the time of injury and visiting time among the respondents. For the purpose of this study, a delay in initiation of PEP was defined as initiation of PEP more than 24 hours after potential rabies virus exposure. The variable was coded as follows: 0 = “prompt PEP”, 1 = “delay of initiation on PEP”. Refused (n = 4) response were excluded.

### Key predictors

#### Potential covariates

Demographic variables included age (1 = “1–14 years old”, 2 = “15–44 years old”, 3 = 45–59 years old”, 4 = “60 and above”), gender, and education (0 = “without college education, 1 = “college education and above”). To our knowledge, family income, instead of individual income, is a more common representative of the affordability of health service. Therefore, we investigated the household income per month (0 = “> 5000 RMB”, 1 = “≤ 5000 RMB”). The habit of playing with animals (0 = “always”, 1 = “occasionally or never”) and time to the nearest RPCs (0 = “> 90 min”, 1 = “≤ 90 min”) were obtained through self-report.

#### Animal injury history

Participants were asked to identify the type of offending animal. Responses were categorized as: 1 = “domestic animal of other people”, 2 = “own domestic animal”, 3 = “roaming animal”, for the purpose of this analysis, offending animals were coded as follows: 0 = “domestic animal” and 1 = “roaming animal”. The World Health Organization (WHO) categorizes the animal bite as Category I (touching/feeding of animals on intact skin), Category II (nibbling of uncovered skin, minor scratches or abrasions without bleeding, licks on broken skin) and Category III (single or multiple transdermal bites or scratches, contamination of mucous membrane with saliva from licks) [27]. The exposure category was recorded according to the guidelines from the WHO. The previous animal injury history was coded as 0 = “one or more times” and 1 = “none”.

#### Knowledge of rabies

Questions regarding knowledge on rabies included: the source of rabies virus transmission, the transmission routes, the incubation period, rabies fatality and whether rabies is preventable.

Responses were coded as: 0 = “wrong answer or don’t know” and 1 = “right answer”.

### Statistics analysis

Results were analyzed to identify the distribution and ratio for each item. The chi-square test was used to determine whether there were significant differences for categorical variables. An univariate analysis was conducted for each factor using a logistic regression model. The factors analyzed were those that affected wound treatment and initiation of PEP: demographic variables, animal injury history and knowledge of rabies. Wound treatment and initiation of PEP were dependent variables. In this analysis, the results were adjusted for affordability (family income per month) and accessibility (the time to the nearest RPC).

Then, multivariate analysis was performed using a forward stepwise logistic regression model including independent variables for wound treatment and initiation of PEP. SPSS Ver. 21.0 (IBM Corp, Armonk, NY, USA) was used for all analyses. For all comparisons, differences were tested with two-tailed tests and *P* values less than 0.05 were considered statistically significant.

## Results

A total of 1,015 victims of animal-bites were investigated. Two thirds of respondents (66.6%) could have an access to the RPC within 30 minutes, and nearly all victims (96.8%) could visit the RPC within 90 minutes, while the remaining 3.2% of participants spent more than 90 minutes to reach the RPC. The main reason for such a long time to seek medical care was that victims did not know the clinics in their communities could supply rabies vaccine. Twenty-one (65.6%) of the victims who did not know the nearest clinics had vaccine delayed the initiation on PEP. These data indicated that the rabies vaccine is accessible for animal bite victims. The physician categorized the animal bite wounds for further management as Category I (3.3%), Category II (41.1%) and Category III (55.6%) according to the WHO classification.

Table 1 shows characteristics of the 1,015 animal bite victims attending the RPCs. Overall, 81.2% of the animal bite victims did not treat their wounds properly, with only 178 (18.8%) victims cleaning their wounds with water and soap or water only, then disinfecting the wound. 35.3% of animal bite victims went to the RPCs more than 24 hours after exposure. Only 14.2% of the participants knew transmission routes and the incubation period. About half (56.7%) of respondents considered rabies as infectious, and 58.8% had knowledge of rabies fatality.

**Table 1:** Factors associated with wound treatment and initiation of PEP.

Factors	Wound treatment					Initiation of PEP				
	Proper treatment		Improper treatment		*P*	Promptly present		Delayed present		*P*
	N	%	N	%		N	%	N	%	
Gender										
Male	59	32.6	367	47.1	<0.001	290	44.3	160	44.9	0.895
Female	14	67.4	413	52.9		364	55.7	196	55.1	
Age classification										
1 ~ 14 years old	17	9.4	129	16.7	0.075	104	16.1	48	13.5	0.001
15~ 44 years old	107	59.4	411	53.2		316	48.9	216	60.8	
45 ~ 59 years old	33	18.3	152	19.4		133	20.6	63	17.7	
60 years old and above	23	12.8	81	10.9		93	14.4	28	7.9	
Educational attainment										
Without College Education	73	40.6	437	56.4	<0.001	366	56.5	178	50	0.055
College and above	107	59.4	338	43.6		282	43.5	178	50	
Family income per month (RMB)										
≤5000	87	48.9	396	51.6	0.560	336	52.2	177	50.6	0.642
>5000	91	51.1	371	48.4		308	47.8	173	49.4	
Time spent to the RPCs										
≤90 min	177	19.1	751	80.9	0.635	629	96.8	346	96.9	1
>90min	4	13.3	26	3.3		21	3.2	11	3.1	
Like to play with animals										
Always	58.0	32	349.0	44.7	0.002	298	45.6	141	39.5	0.064
Occasionally/Never	123	68.0	432	55.3		356	54.4	216	60.5	
Animal injury history										
No previous history	100	55.2	496	63.5	0.025	421	64.4	210	58.8	0.089
Once or more	81	22.1	285	36.5		233	35.6	147	41.2	
Offending animal										
Domestic animal	162	89.5	674	87.0	0.386	550	84.7	324	91	0.004
Roaming animal	19	10.5	101	13.0		99	15.3	32	9	
Knowledge of source of rabies transmission										
Dog, cat	177	97.8	720	92.2	0.003	603	92.2	335	93.8	0.375
Don't know	4	2.2	61	7.8		51	7.8	22	6.2	
Knowledge of route of transmission										
Bite, scratches	38	21.0	143	79.0	0.006	75	11.5	68	19	0.001
Don't know	102	13.1	679	86.9		579	88.5	289	81	
Knowledge of rabies incubation										
1–3 months	38.0	21	143.0	79	0.006	75	11.5	68	19	0.001
Don't know	143	79.0	679	86.9		79	88.5	289	81	
Knowledge of rabies fatality										
Always fatal	128	70.7	443	56.7	<0.001	367	56.1	226	63.3	0.028
Low mortality/Don't know	53	29.3	338	43.3		287	43.9	131	36.7	

Abbreviations: RPCs-Rabies Prevention Clinics

Table 1 shows the breakdown of the two dependent variable results, including differences in animal injury history, knowledge of rabies and other potential covariates. Wound treatment was related to gender (*P* < 0.001), educational attainment (*P* < 0.001), habit of playing with animals (*P* = 0.002), animal injury history (*P* = 0.025), knowledge of source of rabies transmission (*P* = 0.003), knowledge of transmission routes (*P* = 0.006), knowledge of the rabies incubation period (*P* = 0.006) and knowledge of rabies fatality (*P* <0.001). Similarly, the initiation of PEP was associated with age class (*P* = 0.001), offending animal (*P* = 0.004), knowledge of transmission routes (*P* = 0.001), knowledge of the rabies incubation period (*P* = 0.001) and knowledge of rabies fatality (*P* = 0.028).

We then performed a multivariate logistic regression analysis to assess the risk factors associated with improper and delayed PEP. Table 2 displays the relationship between wound treatment as well as the initiation of PEP and the influencing factors. For wound treatment, those who were male (*P* < 0.001), aged 1–14 years (*P* = 0.027), were without college education (*P* < 0.001), liked playing with animals (*P* = 0.003), knew the source of rabies virus transmission (*P* = 0.018), knew the transmission routes (*P* = 0.007), knew the rabies incubation period (*P* = 0.007) and knew that rabies could be fatal (*P* = 0.001) tended to treat the wound improperly. Compared with those who visited RPCs promptly, those who were 15–44 years old (*P* < 0.001), hurt by domestic animals (*P* = 0.010), knew the transmission routes (*P* = 0.001), knew the rabies incubation period (*P* = 0.001) and knew that rabies could be fatal (*P* = 0.019) were more likely to delay the PEP.

**Table 2:** Multivariable analysis of factors relevant to wound treatment and initiation of PEP.

Factors	Improper wound treatment								Delay of initiation on PEP							
	Affordability and accessibility adjusted				Stepwise variables selected				Affordability and accessibility adjusted				Stepwise variables selected			
	OR	95% CI		*P*	OR	95% CI		*P*	OR	95% CI		*P*	OR	95% CI		*P*
Gender (Ref. = Female)																
Male	1.808	1.281	2.552	0.001	1.871	1.318	2.656	0.000	1.108	0.783	1.325	0.893				
Age classification (Ref. = 60 and above)																
1–14 years old	2.149	1.091	4.236	0.027					1.601	0.926	2.768	0.092	1.561	0.898	2.711	0.114
15–44 years old	1.129	0.675	1.888	0.644					2.359	1.483	3.753	0.000	2.324	1.457	3.707	0.000
45–59 years old	1.352	0.740	2.470	0.327					1.624	0.959	2.749	0.071	1.621	0.953	2.759	0.075
Education (Ref. = College education and above)																
Without college education	1.895	1.347	2.666	0.000	1.698	1.203	2.396	0.003	0.782	0.597	1.025	0.075				
Previous animal injury history (Ref. = Yes)																
No	0.722	0.519	1.005	0.054					1.257	0.962	1.643	0.094				
Habit of playing with animals (Ref. = No)																
Yes	1.708	1.206	2.418	0.003	1.554	1.089	2.216	0.015	0.789	0.604	1.030	0.081				
Offending animal (Ref. = roaming animals)																
Domestic animals	0.818	0.486	1.379	0.451					1.751	1.146	2.675	0.010	1.696	1.103	2.608	0.016
Knowledge of source of transmission (Ref. = No)																
Yes	3.465	1.237	9.709	0.018					0.783	0.459	1.334	0.368				
Knowledge of route of transmission (Ref. = No)																
Yes	1.655	1.151	2.380	0.007					1.823	1.275	2.609	0.001				
Knowledge of rabies incubation (Ref. = No)																
Yes	1.655	1.151	2.380	0.007					1.823	1.275	2.609	0.001	1.844	1.279	2.659	0.001
Knowledge of rabies fatality (Ref. = No)																
Yes	1.808	1.268	2.577	0.001	1.577	1.096	2.270	0.014	0.725	0.554	0.949	0.019				

Family income per month and time spend to the Rabies Prevention Clinics were included in the multiple logistic model.

All items (including family income per month and time spend to the Rabies Prevention Clinics) were included in a stepwise model.

Abbreviation: OR = Odds Ratio; 95% CI = 95% Confidence Interval; Ref = Reference.

Table 2 presents the results of the stepwise logistic regression analysis, where the dependent variables were wound treatment and the initiation of PEP. Males (OR = 1.871, 95% CI: 1.318–2.656, *P* < 0.001), those without college education (OR = 1.698, 95%CI: 1.203–2.396, *P* = 0.003), those that had a habit of playing with animals (OR = 1.554, 95% CI: 1.089–2.216, *P* = 0.015), and those with a knowledge of rabies fatality (OR = 1.577, 95% CI: 1.096–2.270, *P* = 0.014) were related to improper wound treatment. For the delay of initiation of PEP, the relevant variables were 15–44 years old (OR = 2.324, 95% CI: 1.457–3.707, *P* < 0.001), hurt by domestic animals (OR = 1.696, 95% CI: 1.103–2.608, *P* = 0.016), and knowledge of the rabies incubation period (OR = 1.844, 95% CI: 1.279–2.659, *P* = 0.001).

## Discussion

As an entirely preventable disease, rabies is a substantial health concern in Asia and Africa, mainly due to unaffordability and inaccessibility of rabies biologics [10, 28]. Luckily, the provision of rabies biologics has been included in the medical insurance settlement, making it available and affordable for residents in China, which is inconsistent with studies reported in less developed countries [10, 29, 30]. In China, basic medical insurance covers most of the cost of rabies vaccine. Corresponding commercial health insurance covers the full cost of rabies vaccine. According to the China Statistical Yearbook 2016, per capita gross domestic product of Chinese people was 8,093 USD [31]. The mean price for one intramuscular dose of rabies vaccine is 14 USD and the vaccine cost index (mean price/gross domestic product per capita*10^4^) was 17.3. According to Hubei Statistical Yearbook 2016, per capita gross domestic product of Wuhan residents was 16,887 USD [32], and the vaccine cost index was 8.3. A previous study reported that people from area where the vaccine cost index was <20 were more likely to receive vaccination against rabies [33]. Therefore, we consider that the cost of rabies vaccine is almost commensurate with both national and local economic development in China.

However, the efficacious design of rabies control strategies is hampered by the relative absence of information, because few studies have focused on the risk factors of improper PEP, specifically in a country with adequate health resources. In the current study, we found that more than four-fifths of animal bite victims applied no or inappropriate wound treatment, and similar findings have been reported in other national and international research [19, 34, 35]. Our analysis revealed that, most (66.6%) respondents could have an access to the RPC within 30 minutes, and nearly all victims (96.8%) could visit the RPC within 90 minutes. Almost all the participants had health insurance that covered the cost of the rabies vaccine. However, approximately one-third of the respondents attended RPCs more than 24 hours following exposure. This is mostly in line with other studies [29, 36, 37], except for one recent study [37], which reported that the average time of attending a health facility was 1–6 hours among Indian rural children. Our study has revealed that gender, educational attainment, habit of playing with animals and knowledge of rabies fatality were associated with wound treatment. In relation to the initiation of PEP, the risk factors were established as age, offending animal and knowledge of the rabies incubation period. Interestingly, this is the first study to explore the determinants for improper and delayed rabies PEP in China.

Males are more likely to treat wounds improperly or do nothing after exposure, which is in line with previous research [9]. This is not surprising because it is documented that men seek help and use health services less frequently than women [38, 39]. It is assumed that several indicators might be involved in men’s decisions, comprising biological, psychological and sociological considerations [38, 39]. The WHO recommends that immediate washing and flushing of wounds for at least 15 minutes with soap and water, or water alone, and disinfection with substances with anti-viral activity is essential after rabies virus exposure [3]. Immediate treatment of all bites and scratches is necessary and important because the virus can remain within the site of the injury for an indefinite period [9]. Disappointingly, the importance and necessity of wound treatment is always underestimated [40, 41], especially by males. Additionally, previous studies reported that males were found to have a higher level of knowledge on rabies [42] but are less likely to take preventive measures [43]. This suggests the need to create awareness education programs about proper wound treatment, especially targeting males.

The participants without college education were independently associated with higher odds of improper wound treatment. As shown in previous studies from Asia and Africa [42, 44], education appears to be the principal determinant of knowledge on rabies. Having lower education was reported to be a predictor of a low level of knowledge [42]. The possible explanation for this is that individuals with a higher education have more access to information, resulting in a better understanding of the characteristics of the zoonosis [45]. Therefore, a public health program to increase awareness of wound treatment in the general population might be beneficial, targeting populations without college education, in particular, the public health information should be simple enough to be easily understood.

Findings of this study indicate that respondents who liked to play with animals treated wounds improperly or did nothing at all. Previous studies reported that individuals who liked playing with pets had higher levels of knowledge of rabies [25,42]. The absence of proper wound treatment might be due to negligence of the wound treatment procedure. Therefore, the awareness-raising campaign should be a priority, particularly to those who like to play with animals.

In our study, participants aged 15–44 years are more likely to postpone the initiation of PEP. This finding is partly in line with a previous study which reported that the working population and the elderly were associated with delay in initiation of PEP [10]. Victims aged 15–44 years are students or the working population, and the curriculum or working schedule might hinder them from consulting RPCs promptly. However, the lack of time could not be the principal reason, because sick leave from schools and employing units is not difficult to take in China. Therefore, the main reason might be unawareness of rabies PEP. Hence, advocacy programs are needed to generate public awareness of prompt rabies PEP to control rabies.

It was important to note that victims injured by domestic animals delayed the initiation of PEP. As shown in a previous study, these people might hold the wrong views that domestic animals might be less risky than roaming animals [46], when actually 99% of human rabies cases are attributed to domestic dogs [8]. The current study suggests a need to create public awareness regarding the necessity of prompt PEP, especially to those bitten or scratched by domestic animals.

The current results also indicate that the animal bite victims who knew the rabies incubation period are inclined to delay the PEP. The incubation period is usually from one to three months. Some respondents mistakenly believed that the incubation period is so long that prompt PEP is not necessary. In fact, immediate PEP is crucial for neutralizing the rabies virus in the bite site before it spreads into the central nervous system [47,48]. This may suggest that the protection effect of prompt PEP needs to be propagated.

The present study highlighted the lack of knowledge and overall poor awareness about rabies, which are the main reasons for improper wound treatment and delay of PEP [49]. A high prevalence of animal bites coupled with poor knowledge and unawareness is a worrisome trend. The main reason is the lack of effective educational outreach at the population level, which has led to disparities in knowledge of appropriate wound treatment and timely PEP following rabies virus exposure [50]. Mucheru [51] reported that people with adequate rabies knowledge were more likely to perform proper health seeking practices (OR = 3.0, 95% CI = 1.4–6.8). The general public should be made aware of vaccination programs, timely PEP and proper wound treatment [35, 52]. Therefore, it is of utmost importance that public awareness is raised about the timely administration of proper PEP [53], which is in line with the theme of the year 2016 World Rabies Day “Educate, Vaccinate, Eliminate”.

### Strengths and limitations of this study

There are several strengths to these analyses that ought to be considered. Firstly, our investigation is the first clinic-based cross-sectional study to investigate the prevalence and determinants of improper wound treatment, as well as the delay in initiation on rabies PEP. Secondly, we obtained one important finding that the main obstacle to rabies PEP is neither accessibility nor affordability, but it is the lack of knowledge and poor awareness. Thirdly, these findings provide a new roadmap to control rabies. The global strategy for rabies prevention and control should be adopted in different regions. For example, vaccines should be available and affordable in less developed countries with the highest rabies disease burdens. Education and awareness-raising programs encourage victims to take proper PEP, and communities should be involved in eliminating rabies at local, national, regional and international levels. This strategy should be administered in all rabies endemic areas, particularly in developed and developing countries.

Unavoidably, this study has some limitations that need to be acknowledged. First, given the limitations of the cross-sectional design, firm conclusions concerning its possible causal effect cannot be drawn. However, the findings can be valuable for providing directed public health messaging and interventions. Second, the study site is mainly in an urban area, with few (1.2%) farmers. However, it has been reported that there was no significant difference in knowledge between participants from urban and semi-urban area [25]. Third, the affordability and availability were proxy-assessed, nevertheless, due to the universal medical insurance and general primary care in China, the rabies vaccine is affordable and available in most areas. Fourth, due to the short survey period and without reliable retrospective data, researchers were unable to explore seasonal changes that existed in animal injury cases or in the delay of PEP.

### Conclusion

In conclusion, the clinic-based study showed that a minority of rabies exposure victims treated their wounds immediately and correctly, and more than one third of them went to RPCs for PEP more than 24 hours after exposure, despite the access of PEP being convenient and affordable. The results indicated that the majority of respondents have neither sufficient knowledge nor sufficient prevention awareness. Therefore, large-scale community-based study is needed to assess the contributing factors of rabies prevention and control. The establishment of systematic and sustained programs to propagate rabies knowledge and to generate public awareness for rabies control is a priority today.

## Supporting information

S1 Questionnaire (XLSX)
